# Supplementary material for: Assessing the efficacy of a job rotation for improving occupational physical and psychosocial work environment, musculoskeletal health, social equality, production quality and resilience at a commercial laundromat: protocol for a longitudinal case study
Source: BMJ Open. 2023 May 12;13(5):e067633. doi: 10.1136/bmjopen-2022-067633 (PMC10186472; doi:10.1136/bmjopen-2022-067633)
Supplement: Author's manuscript [file bmjopen-2022-067633.draft_revisions.pdf]

BMJ Open is committed to open peer review. As part of this commitment we make the peer review history of every article we publish publicly available.

When an article is published we post the peer reviewers' comments and the authors' responses online. We also post the versions of the paper that were used during peer review. These are the versions that the peer review comments apply to.

The versions of the paper that follow are the versions that were submitted during the peer review process. They are not the versions of record or the final published versions. They should not be cited or distributed as the published version of this manuscript.

BMJ Open is an open access journal and the full, final, typeset and author-corrected version of record of the manuscript is available on our site with no access controls, subscription charges or pay-per-view fees (http://bmjopen.bmj.com).

If you have any questions on BMJ Open's open peer review process please email info.bmjopen@bmj.com

BMJ Open

Assessing the efficacy of a job rotation for improving occupational physical and psychosocial work environment, musculoskeletal health, social equality, production quality, and resilience at a commercial laundromat: Protocol for a longitudinal case study.

Journal:	*BMJ Open*
Manuscript ID	bmjopen-2022-067633
Article Type:	Protocol
Date Submitted by the Author:	22-Aug-2022

Complete List of Authors:	Jackson, Jennie; University of Gävle, Department of Occupational Health Sciences and Psychology<br>Sund, Marianne; Elis Textile Service AB<br>Barlari Lobos, Griztko ; Elis Textile Service AB<br>Melin, Lars; Elis Textile Service AB<br>Mathiassen, Svend ; University of Gavle, Occupational Health Sciences and Psychology

Keywords:	OCCUPATIONAL & INDUSTRIAL MEDICINE, HEALTH ECONOMICS, Musculoskeletal disorders < ORTHOPAEDIC & TRAUMA SURGERY

SCHOLARONE™
Manuscripts

Assessing the efficacy of a job rotation for improving occupational physical and psychosocial work environment, musculoskeletal health, social equality, production quality, and resilience at a commercial laundromat: Protocol for a longitudinal case study.

Jennie A. Jackson[a*], Marianne Sund[b], Griztko Barlari Lobos[b], Lars Melin[b], Svend Erik Mathiassen[a]

a. Centre for Musculoskeletal Research, Department of Occupational Health Sciences and Psychology, University of Gävle, 801 76 Gävle, Sweden
b. Elis Textile Service AB, Hamrångevägen 21, 816 31 Ockelbo, Sweden

[*] corresponding author – jennie.jackson@hig.se.

For peer review only - http://bmjopen.bmj.com/site/about/guidelines.xhtml

Abstract

Introduction: Job rotation is a work organization strategy used to reduce work-related exposures and musculoskeletal complaints, yet evidence for the efficacy of the approach is weak. Mismatch between job rotation and company needs, lack of full implementation, lack of exposure variation in included tasks, and failure to assess variation may underlie inconclusive research findings to date. The study aims to develop a job rotation with company stakeholders, perform a process evaluation of the implementation, and determine the extent to which the intervention improves the physical and psychosocial work environment, indicators of health, gender and social equality among workers, and production quality and resilience.

Methods and analysis: Approximately 60 production workers at a Swedish commercial laundromat will be recruited. Physical and psychosocial work environment conditions, health, productivity and gender and social equality will be assessed pre- and post- intervention using surveys, accelerometers, heart rate, electromyography and focus groups. A task-based exposure matrix will be constructed, and exposure variation estimated at the level of the individual worker pre- and post- intervention. An implementation process evaluation will be conducted. Job rotation efficacy will be assessed in terms of improvement in work environment conditions, health, gender and social inequality, and production quality and resilience. This study will provide novel information on the effects of the job rotation on physical and psychosocial work environment conditions, production quality and rate, health, and gender and social inequality among blue-collar workers in a highly multicultural workplace.

Ethics and dissemination: The study received approval from the Swedish Ethical Review Authority. Results of the project will be shared directly with the employees, managers and union representatives from the participating company, other relevant labour market stakeholders, and with researchers at national and international conferences and via scientific publication.

Trial registration: The study is pre-registered with the Open Science Framework (https://osf.io/zmdc8/).

Keywords: repetitive work, variation, work task organization

For peer review only - http://bmjopen.bmj.com/site/about/guidelines.xhtml

Strengths and Limitations

- Co-creation participative intervention and study design
- Assessment of job rotation intervention effects on physical and psychosocial work environment conditions, health, production quality, and resilience with consideration to gender and social equality
- Process evaluation of the intervention implementation
- Consideration of inequality both from a male-female (gender) and a place of birth (ethnicity)
- Case study based on a limited number of workers at a single work site

For peer review only

For peer review only - http://bmjopen.bmj.com/site/about/guidelines.xhtml

Introduction:

Job rotation is an organization-level strategy that involves alternating workers between tasks that, ideally, differ in physical and psychosocial demands. In the field of ergonomics, job rotation has predominantly been used with the aim of reducing work-related exposures and musculoskeletal complaints; however, current evidence has been described as weak (1) or inconclusive (2) regarding the efficacy of job rotation to prevent musculoskeletal disorders (MSD). Recent work has even suggested that job rotation can increase the overall risk of workplace injury (3).

The efficacy of a job rotation is theoretically dependent on the extent to which the tasks included in the rotation differ and thus the extent to which more variation can be introduced in the jobs of individual workers rather than appearing between workers (4). Quantification of exposure *variation* is therefore required before and after job rotation to determine whether the intervention succeeded in altering exposure patterns. Mixed study results to date regarding the effects of job rotation on health outcomes may be the result of a lack of objective assessment of exposure variation both before and after intervention. In Leider's 2015 systematic review of evidence of the effects of job rotation on musculoskeletal complaints, only 4 of the 16 studies quantified aspects of both exposure and MSD health outcomes, and none of the studies assessed exposure variation (5–8).

Consideration of psychosocial factors is also paramount when considering the complex relationships between work organization, work environment and health (including MSDs) (9–13) – Figure 1. Similar to physical exposures, job rotation is generally undertaken to improve psychosocial conditions. While positive correlations have been shown for higher worker job satisfaction ratings (1), there is inconclusive evidence to date for the effects of job rotation on other psychosocial risk factors, for example, job control (14) or health outcomes, including work absence or sick leave (1). Again, mixed study results to date may be the result of low quality study designs and the lack of assessment of exposure and outcome variables before and after intervention.

The conceptual model used in this study is given in Figure 1 and illustrates the interconnection of ergonomic and production factors and outcomes which warrant consideration when making changes in work organization. R*ationalization* is defined here as comprising changes made to *'maximize productivity, quality and efficiency under the prevailing conditions of, among other things legislation, education level of available work force, and culture'* (11). In addition to potential ergonomic gains, job rotation has been shown to improve employee skill/competence, productivity, quality (15,16), decrease worker turnover (15), and improve organizational flexibility (16). Thus, job rotation is *per se* a rationalization. At the time this paper was written, we were unable to find any studies that had simultaneously and quantitatively addressed the effects of a job rotation on the interrelated ergonomics and production factors and outcomes outlined in figure 1. The job rotation will be designed according to the Goldilocks Work approach (17,18) to distribute physical and mental loads during productive work to improve worker health and, to the extent possible, capacity. The job rotation will aim to distribute tasks with higher physical and/or mental demands across workers, and to schedule work to facilitate recovery by interspacing higher demand tasks with lower demand tasks.

insert Figure 1 around here

For peer review only - http://bmjopen.bmj.com/site/about/guidelines.xhtml

Inconclusive findings in previous job rotation studies may also be the result of a mismatch between specific company needs and the implemented job rotation (19,20). To improve intervention efficiency, a participatory approach has been called for in which interventions are co-created by researchers together with relevant company stakeholders, such as, management, health and safety, union, and production representatives (21,22). Co-creation can also increase knowledge within an organization regarding the problem(s) to be targeted by an intervention and how the intervention will address the problem(s). This can enhance readiness for change, which is a necessity for successful intervention implementation (23,24). Co-created program logic (COP) is a participatory approach that begins with a set of outcomes identified by stakeholders, around which a specific intervention is subsequently designed (25). COP is also a tool for defining, through negotiation between stakeholders and researchers, the outcomes to be measured and the optimal time points for the measurements. COP has proved successful in developing study aims and design and in improving readiness for change, intervention contextual fit, and implementation efficacy (23).

Insufficient process evaluation of the intervention implementation, including failure to fully implement the intervention (26), has also been identified as a common weakness in prior job rotation studies (27). Consideration of how job rotation has been implemented in sub-populations of workers may prove salient in fully understanding the implementation and in the effects of an intervention, but to date has been sparse. Findings from a job rotation intervention study at a grocery store found work tasks deemed worst by employees were excluded from the intervention and subsequently allotted to a group of female workers who were, thus, not included in the job rotation (28). This example is in line with the findings of a 2013 review report prepared for the Swedish Work Environment Authority, finding that task allocation within jobs was gendered, where female workers tended to be given more monotonous and repetitive tasks than male workers (29). Further, there is broad consensus in the literature that females receive less on-the-job training than men, including initial on-the-job training (30), which positions females to be assigned fewer tasks and possibly to be excluded for job rotation initiatives comprising tasks requiring more than basic training or competences. Foreign-born employees have also been shown to receive less on-the-job training than native born employees (31). Consideration of job rotation design and implementation within a social context may therefore be crucial. The many limitations of previous studies and the lack of research simultaneously considering aspects of social inequality in task assignment, job rotation, and health demonstrate the need for further research that considers the effects of job rotation from a wide range of perspectives.

For peer review only

Methods and analysis

The aim of this study is to develop a job rotation intervention at a commercial laundromat in concert with company stakeholders using co-created program logic and to determine the extent to which the intervention can improve the physical and psychosocial work environment and indicators of health, advance gender and social equality among workers, and increase production quality and resilience without negatively affecting production rates.

The specific aims of the study are to:

1) Describe the co-creation program logic process used to develop the intervention and study design.
2) Provide a process evaluation for the implementation, including assessment of readiness for change and the extent to which the intervention was implemented.

For peer review only - http://bmjopen.bmj.com/site/about/guidelines.xhtml

3) Determine the extent to which the job rotation intervention increased exposure variation and lead to changes in physical and mental work environment factors and indicators of health.
4) Evaluate social and gender inequality in work organization, working conditions, and health before, during, and after the job rotation intervention
5) Assess the impact of the job rotation on production rate, quality and resilience.

Co-created program logic

The study will be performed at a commercial laundromat employing approximately 50 full time workers in laundry handling tasks. The study resulted from contact initiated by the study site: a commercial laundromat in Sweden. The laundromat management and union saw room for improvement in physical and psychosocial working conditions and issues of equality, and were interested in implementing a job rotation, but sought help to successfully make the change. We began by establishing a steering committee of eight people that comprised company management (LM, GBL), production workers, health and safety and union representatives (MS) and researchers (JJ, SEM) to jointly develop the study. Using co-created program logic (COP), outcome metrics were established by the researchers in concert with the steering committee using an iterative procedure based on identifying desired changes that could directly be tied to the job rotation intervention. The study scientific testing protocol was then developed (JJ, SEM) and presented to the steering committee who completed a risk analysis prior to the protocol being approved.

The job rotation intervention *per se* was also co-created in a collaboration between management (GBL), union representatives (MS) and researchers (JJ), and was guided by the Goldilocks Work approach principals (17,18) to find a health-promoting balance in both physical and mental work exposures. To better understand laundry processing tasks, one researcher (JJ) shadowed workers performing each task and, to the extent possible, spent 15-45 minutes performing each task. Company representatives and the researcher worked together to rate the physical and mental load (heavy/light) and the degree of repetitiveness (monotonous/variable) of each task. Led by the research team, company representatives grouped tasks into different proficiency levels, starting with a base group of tasks that they believed all workers could and should learn, and then moving onto tasks that required increased knowledge of production flow and task-specific training. Five hierarchical levels of tasks were created, namely: (i) basic tasks (which all workers could perform), (ii) driers and small machines, (iii) order picking and packing for hotel and medical customers, (iv) order picking and packing of work clothes, and (v) steering production flow.

The laundromat is physically divided into two sides – the *sorting* side, where bags of dirty laundry are received, emptied onto conveyor belts, and goods are sorted by colour and item prior to washing, and the *processing* side of the laundromat, where clean goods are dried, pressed, folded and packed. In general, rotation from sorting to processing is not typically done within a day as a shower is required before moving from sorting to processing. Base tasks were identified on both the sorting and processing sides, and so full-day rotation plans were established for each side to accommodate the hygiene requirements.

To meet company goals for improved language proficiency, social and gender equality and integration, the job rotation was based on *teams* comprising 3-6 workers that rotated as a unit. Teams were created to include at least 1 male and 1 female, 1 native Swede, ideally not more than 1 person with the same mother-tongue, and a mix of seniority levels in the competencies required for the tasks assigned to the team. A total of ten job rotation teams were formed; five of which performed basic tasks. Of these, four teams were assigned both sorting work and processing work,

For peer review only - http://bmjopen.bmj.com/site/about/guidelines.xhtml

while one base group was assigned only processing work. Job training was required for nearly all workers to be able to perform all tasks assigned under the job rotation intervention. The company aimed to, in parallel, develop a new salary structure that would match the new proficiency 'steps' and competencies with employee wages. The company expected that the new work and wage structure would make for a more transparent development and promotion path, including making it easier for employees to understand and express interest in career advancement plans.

Study design overview

The workers will be followed for approximately 12 months, with measurements occurring prior to, during, and 8-12 months following the job rotation intervention implementation (Figure 2). Baseline physical and psychosocial working conditions will be documented using (i) a questionnaire to assess the physical and psychosocial environments, (ii) technical measurements to assess posture, heart rate, and muscle activity levels during work, and (iii) semi-structured interviews to assess readiness for change. Initial response to the intervention will be assessed six weeks post implementation via semi-structured focus group interviews. At follow up, physical and psychosocial working conditions will be re-assessed using the baseline measurement protocol, and a final set of semi-structured focus group interviews will be conducted to document production and working environment changes that occurred during the follow-up period but were unrelated to the job rotation intervention (32). Production rate and quality will be assessed weekly throughout the study period, using data collected by the company.

insert figure 2 around here

Study population

Permanent and contract workers currently employed in laundry handling tasks at the laundromat will be invited to participate in the study; the exact number of participants and inclusion criteria will vary by measurement type and research question, as outlined below. For the questionnaire, we will aim for a response rate of 70% (n = 35), and we will aim for 15 participants in both the technical measurement protocols. Focus groups interviews will be conducted with each team and will include all team members who elect to participate.

Approximately half of the employees are foreign born with varying levels of Swedish language proficiency, and there is a wide range in education levels across all employees. To ensure workers are informed, understand participation is voluntary and understand what participation entails, we will present all aspects of the research project in person to all employees using slides relying primarily on graphical displays of information, and we will provide live demonstration of technical measurement methods.

All production employees will be invited to participate in the study. Baseline survey response rates were 97% (n = 58), which suggests a strong likelihood for obtaining ample data for this case study throughout the follow-up period. Focus on developing and maintaining a strong relationship with the company has been present since study inception.

For peer review only - http://bmjopen.bmj.com/site/about/guidelines.xhtml

Pre- and post- job rotation measurements

Questionnaire

A custom questionnaire comprised of modules from well-established, validated and documented questionnaires and modules adapted from questionnaires previously used in our research group to assess other occupational groups (ex. (33,34) but adjusted to specifically capture information about the commercial laundromat environment. The modules in our questionnaire consider: personal and demographic parameters (9 questions), employment status and working hours (7 questions), specific work tasks currently performed and time spent per task over a week (6 questions), physical and mental demands of each work task and the overall job (4 questions with Borg CR-10 response scale (35)), self-rated work ability (4 questions), workload, fatigue and recovery (short SOFI questionnaire (36,37), 14 questions); musculoskeletal pain (Standardised Nordic questionnaire (38), 4 questions), job satisfaction (2 questions), and the psychosocial work environment (selected Copenhagen Psychosocial Questionnaire (COPSOQ) modules (39), 79 questions).

All employees will be invited to complete the questionnaire. The language and education level of the respondents was a focus during the development of all questionnaire modules, and substantial efforts were made to simplify language and response options in all custom modules. Where appropriate, a colour bar, ranging from green to red, was added to help participants understand the value (positive/neutral/negative) of the response options (figure 3a). We also added a colour bar to each COPSOQ question which helped to highlight that the response scales vary (positive to negative or negative to positive) by question (figure 3b).

insert figure 3 around here

To further assist employees, a workplace language course will be given one month prior to introducing the questionnaire for the 24 employees deemed to have the lowest language proficiency. As part of the course, a Swedish language teacher will review the questionnaire and teach employees how to respond to the different types of questions (short answer, multiple choice and CR-10 response scale). Lessons will be provided during working hours for groups of 8 employees at a time, be 1 hour in duration, and occur twice a week. In addition, all employees will be provided with a copy of the questionnaire one week in advance of the data collection.

Questionnaires will be completed during paid working hours in small groups (6-8 employees) sitting in the same room as a researcher. We will provide help as needed and encourage workers to join a group with other workers sharing the same mother tongue. Mobile phones and translation/dictionary apps will be permitted and encouraged. Workers will also be permitted to answer the survey at home if desired. The on-site researcher will have trained in giving consistent explanations. The same researcher will be present on all occasions.

Technical Measurements

Pre-implementation, technical data collection will be conducted to assess postures and heart rate over five consecutive days, and then to assess muscle activity levels during individual work tasks. All full-time permanent and contract employees working day shifts will be invited to participate in one or both technical data collections.

For peer review only - http://bmjopen.bmj.com/site/about/guidelines.xhtml

Post-implementation, we will evaluate any work tasks that have changed and any new work tasks that have been introduced.

Posture and heart rate assessment

Working postures, heart rate and heart rate variability will be assessed continuously over five consecutive working days. Postures will be assessed using wireless tri-axial accelerometers (Axivity, Newcastle upon Tyne, UK) affixed to the skin using double sided toupee tape (between the device and the skin) and breathable medical tape (over top of the device). Upper arm elevation will be recorded using units mounted according to 'LT INC' location (40): upper edge of the device aligned with the insertion of the deltoid muscle into the humerus, long axis aligned with the humerus. Trunk elevation angle will be recorded using a unit placed on the sternum with the top edge of the unit at the level of the sternoclavicular notch, and the long axis of the unit aligned with the line of gravity. A final accelerometer unit will be placed on the right thigh (long axis aligned with the femur) and will be used to determine activities (sit/stand/walk/run) during the five working days.

During the same 5-day period, participants will also wear a BodyGuard heart rate (HR) monitor (Firstbeat Technologies Oy, Finland) comprising two electrodes (Ambu, Denmark) positioned according to Firstbeat recommendations, a wireless logger (attached to the electrode positioned directly below the right clavicle), and a wire joining the two electrodes.

To permit task-level analysis of posture and heart rate, workers will record the specific tasks and times at which each task was performed over the 5-day period in a daily log book. A researcher will briefly meet each worker individually once a day during the 5-day period. Placement and alignment of all accelerometer units will be verified and tape changed, as necessary, depending on worker discomfort and tape adhesion, and calibration exercises will be performed according to (40). The researcher will also confirm that the participant has been completing the work task log and responding to the fatigue assessment questions.

Muscle activity assessment

Muscle activity will be assessed using electromyography (EMG) recorded continuously during one working bout of approximately 2 hours, and during the adjacent coffee break (15 minutes) or lunch break (30 minutes). During a work bout, workers typically perform one task, but may perform two or more tasks. EMG will be recorded bilaterally from the trapezius (Tr EMG) and forearm (FA EMG) muscles using a portable logger and EMG sensors from Biometrics Ltd. (Newport, UK). At each recording site, the skin will be shaved and cleaned with alcohol prior to applying a disposable electrode (Ambu, Denmark). A single ground electrode (Ambu, Denmark) will be positioned atop a prominent thoracic vertebra (usually C7). Tr EMG electrodes will be positioned 2 cm lateral to the midpoint between C7 and the acromion process and aligned along the direction of the muscle fibres (41). Bilaterally, a 'through the forearm' assessment of FA EMG will be made, as described by (42), using electrodes placed just lateral to the muscle bellies of the flexor digitorum superficialis and extensor digitorum communis muscles. Given the physical nature of the laundry handling work tasks, the often tight spaces through which workers navigate, and the heavy machinery involved in the work, care will be made to ensure the EMG recording equipment is securely attached to the participant and that the cables will not interfere with their work.

A rest file and three repeats of muscle-specific reference contractions will be collected to permit normalisation. For the trapezius muscles, a sub-maximal reference voluntary exertion (RVE) normalisation will be employed according to (Mathiassen, et al., 1995). RVE trials will be 15 s in

For peer review only - http://bmjopen.bmj.com/site/about/guidelines.xhtml

duration (Åkesson et al., 1997; Hansson et al., 2000) interspaced by 30 s of rest. For the forearm, a maximum voluntary exertion (MVE) pinch grip normalisation task will be employed. Subjects will gradually increase their pinch grip on a pinch dynamometer over 3 seconds, until reaching and holding their MVE for 3 seconds before gently ramping down their exertion. MVE trials will be approximately 7 s in duration interspaced by 60 s of rest

A researcher will follow workers during the EMG recording period to document the times and tasks performed. Workers will also be videotaped during the EMG recording period to provide additional assistance in dividing the full EMG recording into component tasks.

Focus group interviews

Semi-structured focus group interviews will be conducted with each job rotation team and will occur at three time points. A researcher from our research group who is not otherwise involved with the project will conduct all interviews.

Immediately prior to implementation, interviews will be used to assess readiness to change and workers' first impressions of their new working schedules. At 6-weeks post implementation, interviews will be used to document the extent of implementation during the initial phase, capture initial facilitators and barriers to implementation, and document the self-reported ability of workers to complete all relevant tasks. At follow up (8-12 months post implementation), interviews will be used to document the extent of implementation achieved during the study, capture facilitators and barriers to the intervention, and to document changes that occurred during the follow-up period that were un-related to the job rotation intervention, but may have affected the way in which work was performed.

Company data

The laundromat will track and provide weekly data on production rate (total tonnes laundered), quality, worker sick days, days off for workers to care for sick children, accidents, injuries and worker turnover from November 2019 until the end of the follow up period

Data processing and statistical analysis

Most previous studies documenting working conditions and occupational physical exposures via questionnaires and technical measurements have data analyses based on standard statistical methods, including a priori power calculations. Such methods assume that participants represent, in theory, the total population of all workers in similar settings. In our study, results consider a specific workplace and its 50-60 participant workers and not all workers employed in all commercial laundromats. We accept this notion in the research project and acknowledge it by adjusting basic descriptive statistics, such as SDs, by a finite population correction term (34).

Questionnaire data

Differences pre- to post- job rotation in work task allocation, psychosocial work environment conditions (including COPSOQ dimension) and self-reported health will be analysed, including consideration of confounders such as age, geographical location, marital status, family situation and

For peer review only - http://bmjopen.bmj.com/site/about/guidelines.xhtml

other socioeconomic factors ultimately included in the questionnaires. Change scores will be calculated between baseline and follow-up for all COPSOQ dimensions.

The extent to which factors differed between males and females and between Swedish-born and foreign-born employees will, to the extent possible, be considered using a stratification approach.

Technical measurement data

Posture and heart rate assessment

The Firstbeat SPORTS uploader (V.1.0; Firstbeat Technologies) will be used for uploading and visually identified artefacts will be removed. Average heart rate (HR) during work, non-work and sleep will be calculated using a custom MATLAB script. Relative aerobic workload will be calculated in a previously developed custom MATLAB script and will be based on the heart rate reserve (HRR), which is a well-established estimate of the body's workload and the body's workload relative to individual fitness.

The accelerometry data will be processed using a custom-made MATLAB program to determine the time spent sitting, standing, walking, in light physical activity and in moderate to vigorous physical activity during both working- and non-working hours. Trunk and upper arm elevation data will be calibrated (40) to angles calculated using an in-house custom MATLAB program.

Accelerometry and HR data will be determined for each individual work task using the log book kept by workers. For each task, HR variables calculated will include HR intensity (relative aerobic workload, measured in terms of % HRR), time at different intensity levels, and HR variability, and postural variables, mean angle, time in neutral, and minute-to-minute variance of angle (43). Tasks where worker HR reaches or exceeds 60% HRR will be deemed sufficiently high-intensity for potentially generating increases in physical capacity (44).

Muscle activity assessment

Post collection, EMG signals will be Butterworth filtered (30 Hz high pass) for minimization of electrocardiogram (ECG) contamination (Drake and Callaghan, 2006), offset corrected, and root-mean-square (RMS) converted (100 ms moving window), then quadratically rest adjusted. All files will be visually inspected to ensure good data quality. The mean amplitude across a stable 10s period from each of the three RVE trials will be calculated and the mean of the three trials will be used to normalise the subject's Tr-EMG data. The largest 0.5 s mean EMG value will be determined across each MVE, and the largest single MVE will be used to normalise the subject's FA-EMG

EMG data from the approximately 2-hour work bout will be partitioned, if necessary, to individual work tasks using the time log kept by the researcher during data collection and, when necessary, assisted by the time stamp in the video data. For each task, the following muscle activity variables will be calculated using a custom-made MATLAB program: mean, peak, time at rest, minute-to-minute variance, and SULMA periods (45).

Task-based exposure matrix and estimates of variation

A task-based exposure matrix will be constructed comprising the posture and muscle activity variables, as outlined above. Using the log book data, individual exposure profiles will be constructed by estimating exposure variation for each day using an approach similar to the job variance ratios described by (43). Weekly variation will be assessed at the individual level by the mean variation across days and the between-days variance across the five days.

For peer review only - http://bmjopen.bmj.com/site/about/guidelines.xhtml

Pre-post changes in individual estimates of daily and weekly exposure variation will be assessed using a repeated measures ANOVA.

Interview data

Group semi-structured focus-group interview data will be transcribed and analysed thematically (46), guided by research aim 2 to assess readiness for change and the extent of intervention implementation. To the extent possible, differences in experiences of the job rotation will be considered between male and female employees and Swedish- and foreign-born employees. Management interview data will be primarily used to assist in interpreting and understanding survey and technical data findings including changes occurring during the follow-up that could have influenced work organization or work environment unrelated to the intervention (32)(ref).

In interpreting focus group interviews Acker's theory of inequality regimes will be used as a framework for assessing how inequalities are produced and reproduced (47). Acker's theory comprises both organizational and individual levels and states that inequality regimes exist in all organizations, and can be defined as 'loosely interrelated practices, processes, actions and meanings' that create power orders from intersections of gender and ethnicity within the organization (47).

Registration

The study is pre-registered with the Open Science Framework (https://osf.io/zmdc8/).

Ethics and dissemination

The study received approval from the Swedish Ethical Review Authority (reference number 2019-00228). Written informed consent will be obtained for each data collection method, namely: accelerometry and heart rate monitoring, EMG, survey and focus group interview participation.

The results of the project will be shared directly with the Elis employees and managers at the participating laundromat, the worker's union (IF Metall) and the central Swedish and Global Elis-concerns via presentations and summary reports. Research results will also be disseminated via publication in international, peer-reviewed, open-access scientific journals and presentation at international scientific conferences.

Strengths and Limitations

The major novel contributions of this study to the literature are (i) the broad scope of the evaluation of the job rotation intervention, including consideration of the effects of the rationalization on work environment conditions, health, and production quality and resilience with consideration to gender and social equality and (ii) the inclusion of a process evaluation of the intervention implementation.

The consideration of inequality both from a male-female (gender) and a place of birth (ethnicity) perspective is in line with intersectionality theory (48) and further reflects the reality of modern immigration. It also reflects the Swedish government's latest Work Environment Strategy which prioritizes increased efforts towards sustainability with the goal of all employees (regardless of sex or place of birth) having a work environment that makes them able to, capable of, and willing to work until official retirement age (49).

The primary limitation of the study is the limited number of workers, and the inclusion of a single work site which will limit the generalizability of the results. Still, we anticipate that our findings on

For peer review only - http://bmjopen.bmj.com/site/about/guidelines.xhtml

the impact of job rotation on physical and psychosocial work conditions, health and equality will be immediately useful for other laundromats in the global Elis concern. Further, we anticipate findings regarding social equality to be relevant for other commercial laundromats and small- and medium-sized production-based industries with high employee diversity.

Funding

This work was supported by the Swedish Research Council for Health, Working Life and Welfare (Forte) grant number 2009-1761.

Authors' contributions

JJ and SvE conceptualized the project. All co-authors participated in the co-creation process in which the study design and intervention were developed with JJ leading the process. All authors made substantial contributions to the development of the research study aims. JJ, MS and GBL developed the job rotation. JJ and SEM developed the research study design. JJ collected pilot data to assess task exposure variation.

All authors approve the submitted version and agree to be personally accountable for their contributions.

All authors read and approved the final manuscript

Acknowledgements

The authors wish to thank the production workers at the Elis laundromat in Ockelbo Sweden for generously welcoming the research team and embarking on this journey together with genuine enthusiasm.

Competing interests statement

The authors declare no competing interests.

For peer review only - http://bmjopen.bmj.com/site/about/guidelines.xhtml

Figure Titles

Figure 1: Conceptual model illustrating overlapping interests of production and ergonomics. *Proposed interactions are shown in arrows between rationalization (job rotation, in the current study) and work environment factors (shown in rectangles), and health and productivity outcomes (shown in ovals). Model adapted from* (11,12).

Figure 2: Study design

Figure 3: Examples of survey questions showing colour bar used to assist in understanding the value of response options. *(a) A custom module question and (b) A COPSOQ question.*

For peer review only - http://bmjopen.bmj.com/site/about/guidelines.xhtml

References

1. Padula RS, Comper MLC, Sparer EH, Dennerlein JT. Job rotation designed to prevent musculoskeletal disorders and control risk in manufacturing industries: A systematic review. Appl Ergon [Internet]. 2017;58:386–97. Available from: http://dx.doi.org/10.1016/j.apergo.2016.07.018

2. Leider PC, Boschman JS, Frings-Dresen MHW, van der Molen HF. Effects of job rotation on musculoskeletal complaints and related work exposures: a systematic literature review. Ergonomics. 2015;58(1):18–32.

3. Kuijer PPFM, Van Der Beek AJ, Van Dieën JH, Visser B, Frings-Dresen MHW. Effect of job rotation on need for recovery, musculoskeletal complaints, and sick leave due to musculoskeletal complaints: A prospective study among refuse collectors. Am J Ind Med. 2005;47(5):394–402.

4. Mathiassen SE. Diversity and variation in biomechanical exposure: What is it, and why would we like to know? Appl Ergon. 2006;37(4 SPEC. ISS.):419–27.

5. Fernström EAC, Åborg CM. Alterations in shoulder muscle activity due to changes in data entry organisation. Int J Ind Ergon. 1999;23(3):231–40.

6. Fredriksson K, Bildt C, Hägg G, Kilbom Å. The impact on musculoskeletal disorders of changing physical and psychosocial work environment conditions in the automobile industry. Int J Ind Ergon. 2001;28(1):31–45.

7. Rissén D, Melin B, Sandsjö L, Dohns I, Lundberg U. Psychophysiological stress reactions, trapezius muscle activity, and neck and shoulder pain among female cashiers before and after introduction of job rotation. Work Stress. 2002;16(2):127–37.

8. Arvidsson I, Balogh I, Hansson G åke, Ohlsson K, Åkesson I, Nordander C. Rationalization in meat cutting - Consequences on physical workload. Appl Ergon. 2012;

9. Bao SS, Kapellusch JM, Merryweather AS, Thiese MS, Garg A, Hegmann KT, et al. Relationships between job organisational factors, biomechanical and psychosocial exposures. Ergonomics. 2016;59(2):179–94.

10. Bongers PM, Kremer AM, Laak J Ter. Are psychosocial factors, risk factors for symptoms and signs of the shoulder, elbow, or hand/wrist?: A review of the epidemiological literature. Am J Ind Med. 2002;41(5):315–42.

11. Winkel J, Westgaard RH. Editorial: A model for solving work related musculoskeletal problems in a profitable way. Appl Ergon. 1996;27(2):71–7.

12. Rolander B, Jonker D, Winkel J, Sandsjö L, Balogh I, Svensson E, et al. Working conditions, health and productivity among dentists in Swedish public dental care - a prospective study during a 5-year period of rationalisation. Ergonomics. 2013;56(9):1376–86.

13. Sorensen G, McLellan DL, Sabbath EL, Dennerlein JT, Nagler EM, Hurtado DA, et al. Integrating worksite health protection and health promotion: A conceptual model for intervention and research. Prev Med (Baltim) [Internet]. 2016;91:188–96. Available from: http://dx.doi.org/10.1016/j.ypmed.2016.08.005

14. Balogh I, Ohlsson K, Hansson GÅ, Engström T, Skerfving S. Increasing the degree of automation in a production system: Consequences for the physical workload. Int J Ind Ergon. 2006;36(4):353–65.

15. Jorgensen M, Davis K, Kotowski S, Aedla P, Dunning K. Characteristics of job rotation in the

For peer review only - http://bmjopen.bmj.com/site/about/guidelines.xhtml

Midwest US manufacturing sector. Ergonomics. 2005;48(15):1721–33.

16. Cristini A, Pozzoli D. Workplace practices and firm performance in manufacturing: A comparative study of Italy and Britain. Int J Manpow. 2010;31(7):818–42.

17. Straker L, Mathiassen SE, Holtermann A. The "Goldilocks Principle": designing physical activity at work to be "just right" for promoting health. Br J Sports Med. 2018;52(13):818–9.

18. Holtermann A, Mathiassen SE, Straker L. Promoting health and physical capacity during productive work: The goldilocks principle. Scand J Work Environ Heal. 2019;45(1):90–7.

19. Moore GF, Evans RE. What theory, for whom and in which context? Reflections on the application of theory in the development and evaluation of complex population health interventions. SSM - Popul Heal [Internet]. 2017;3(December 2016):132–5. Available from: https://doi.org/10.1016/j.ssmph.2016.12.005

20. Nielsen K, Randall R. Opening the black box: Presenting a model for evaluating organizational-level interventions. Eur J Work Organ Psychol. 2013;22(5):601–17.

21. Leask CF, Sandlund M, Skelton DA, Altenburg TM, Cardon G, Chinapaw MJM, et al. Framework, principles and recommendations for utilising participatory methodologies in the co-creation and evaluation of public health interventions. Res Involv Engagem [Internet]. 2019;5(1):2. Available from: https://doi.org/10.1186/s40900-018-0136-9

22. Von Thiele Schwarz U, Nielsen K, Edwards K, Hasson H, Ipsen C. How to design , implement and evaluate organizational interventions for maximum impact : the Sigtuna Principles. Eur J Work Organ Psychol [Internet]. 2021;30(3):415–27. Available from: https://doi.org/10.1080/1359432X.2020.1803960

23. Cedstrand E, Alvesson HM, Augustsson H, Bodin T, Bodin E, Nyberg A, et al. Co-creating an occupational health intervention within the construction industry in Sweden: Stakeholder perceptions of the process and output. Int J Environ Res Public Health. 2021;18(24).

24. Billsten J, Fridell M, Holmberg R, Ivarsson A. Journal of Substance Abuse Treatment Organizational Readiness for Change (ORC) test used in the implementation of assessment instruments and treatment methods in a Swedish National study. J Subst Abuse Treat [Internet]. 2018;84:9–16. Available from: https://doi.org/10.1016/j.jsat.2017.10.004

25. von Thiele Schwarz U, Richter A, Hasson H. Getting everyone on the same page: Co-created program logic (COP). In: Nielsen K, Noblet A, editors. Organizational Interventions for Health and Well-being A Handbook for Evidence-Based Practice. First. Exeter, Devon: Routledge; 2018. p. 42–67.

26. Murta SG, Sanderson K, Oldenburg B. Process evaluation in occupational stress management programs: A systematic review. Am J Heal Promot. 2007;21(4):248–54.

27. Rasmussen CDN. Is job rotation effective for prevention of work-related musculoskeletal disorders and sick leave? Occup Environ Med. 2017;74(8):543–4.

28. Johansson K, Fältholm Y, Abrahamsson L. Job Rotation Meets Gendered and Routine Work in Swedish Supermarkets. NORA - Nord J Fem Gend Res. 2015;23(2):109–24.

29. Lewis C, Mathiassen S. Belastning, genus och hälsa i arbetslivet. Rapport 2013:9. Stockholm; 2013.

30. Grönlund A. On-the-Job Training - A Mechanism for On-the-Job Training Investments Segregation ? Examining the Relationship between Gender , Occupation , and On-the-Job Training Investments. Eur Sociol Rev. 2012;28(3):408–20.

For peer review only - http://bmjopen.bmj.com/site/about/guidelines.xhtml

31. Barrett A, McGuinness S, O'Brien M, O'Connell P. Immigrants and Employer-provided Training. J Labor Res. 2013;34(1):52–78.

32. Edwards K, Winkel J. A method for effect modifier assessment (EMA) in ergonomic intervention research. Appl Ergon [Internet]. 2018;72(May):113–20. Available from: https://doi.org/10.1016/j.apergo.2018.05.007

33. Bergsten EL, Mathiassen SE, Vingård E. Psychosocial work factors and musculoskeletal pain: A cross-sectional study among swedish flight baggage handlers. Biomed Res Int. 2015;2015.

34. Mathiassen SE, Bolin M, Olofsdotter G, Johansson E. Equal health at work? Protocol for an observational study of work organisation, workload and musculoskeletal complaints among women and men in grocery retail. BMJ Open. 2020;10(1):e032409.

35. Borg G. Psychophysical bases of perceived exertion. Med Sci Sport Exerc. 1982;14:377–81.

36. Åhsberg E. Dimensions of fatigue in different working populations. Scand J Psychol [Internet]. 2000 Sep 1;41(3):231–41. Available from: https://doi.org/10.1111/1467-9450.00192

37. Mathiassen SE, Hallman DM, Lyskov E, Hygge S. Can cognitive activities during breaks in repetitive manual work accelerate recovery from fatigue? A controlled experiment. PLoS One. 2014;9(11).

38. Kuorinka I, Jonsson B, Kilbom A, Vinterberg H, Biering-Sørensen F, Andersson G, et al. Standardised Nordic questionnaires for the analysis of musculoskeletal symptoms. Appl Ergon [Internet]. 1987;18(3):233–7. Available from: http://www.sciencedirect.com/science/article/pii/000368708790010X

39. Pejtersen JH, Kristensen TS, Borg V, Bjorner JB. The second version of the Copenhagen Psychosocial Questionnaire. Scand J Public Health. 2010;38(SUPPL. 3):8–24.

40. Jackson JA, Mathiassen SE, Wahlström J, Liv P, Forsman M. Is what you see what you get? Standard inclinometry of set upper arm elevation angles. Appl Ergon. 2015;47:242–52.

41. Mathiassen SE, Winkel J, Hägg GM. Normalization of surface EMG amplitude from the upper trapezius muscle in ergonomic studies—a review. J Electromyogr Kinesiol. 1995;5(4):197–226.

42. Takala EP, Toivonen R. Placement of forearm surface EMG electrodes in the assessment of hand loading in manual tasks. Ergonomics. 2013;56(7):1159–66.

43. Barbieri DF, Srinivasan D, Mathiassen SE, Nogueira HC, Oliveira AB. The ability of non-computer tasks to increase biomechanical exposure variability in computer-intensive office work. Ergonomics [Internet]. 2015;58(1):50–64. Available from: http://dx.doi.org/10.1080/00140139.2014.965753

44. Gormley SE, Swain DP, High R, Spina RJ, Dowling EA, Kotipalli US, et al. Effect of intensity of aerobic training on VO 2max. Med Sci Sports Exerc. 2008;40(7):1336–43.

45. Østensvik T, Veiersted KB, Nilsen P. A method to quantify frequency and duration of sustained low-level muscle activity as a risk factor for musculoskeletal discomfort. J Electromyogr Kinesiol [Internet]. 2009;19(2):283–94. Available from: http://dx.doi.org/10.1016/j.jelekin.2007.07.005

46. Braun V, Clarke V. Qualitative Research in Psychology Using thematic analysis in psychology Using thematic analysis in psychology. Qual Res Psychol [Internet]. 2006;3(2):77–101. Available from: http://www.tandfonline.com/action/journalInformation?journalCode=uqrp20%5Cnhttp://www.tandfonline.com/action/journalInformation?journalCode=uqrp20

For peer review only - http://bmjopen.bmj.com/site/about/guidelines.xhtml

47. Acker J. Inequality regimes: Gender, class, and race in organizations. Gend Soc. 2006;20(4):441–64.

48. Mullings L, Schulz AJ. Gender, race, class, and health: Intersectional approaches. Jossey-Bass; 2006.

49. Regeringen. En god arbetsmiljö för framtiden –regeringens arbetsmiljöstrategi 2021–2025. Skr. 2020/21:92. Stockholm; 2021.

For peer review only

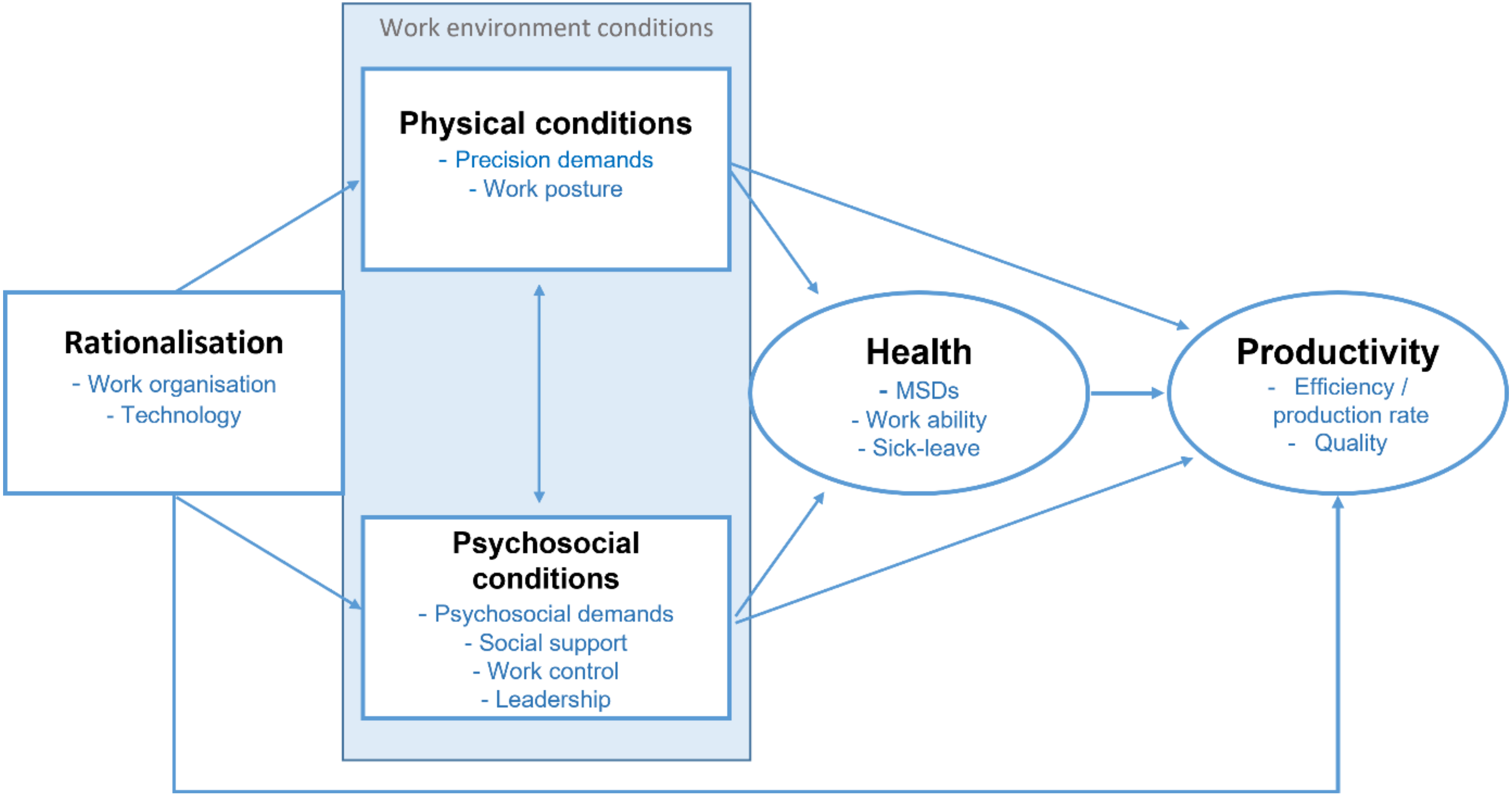
Work environment conditions
Physical conditions
- Precision demands
- Work posture
Rationalisation
- Work organisation
- Technology
Psychosocial conditions
- Psychosocial demands
- Social support
- Work control
- Leadership
Health
- MSDs
- Work ability
- Sick-leave
Productivity
- Efficiency / production rate
- Quality

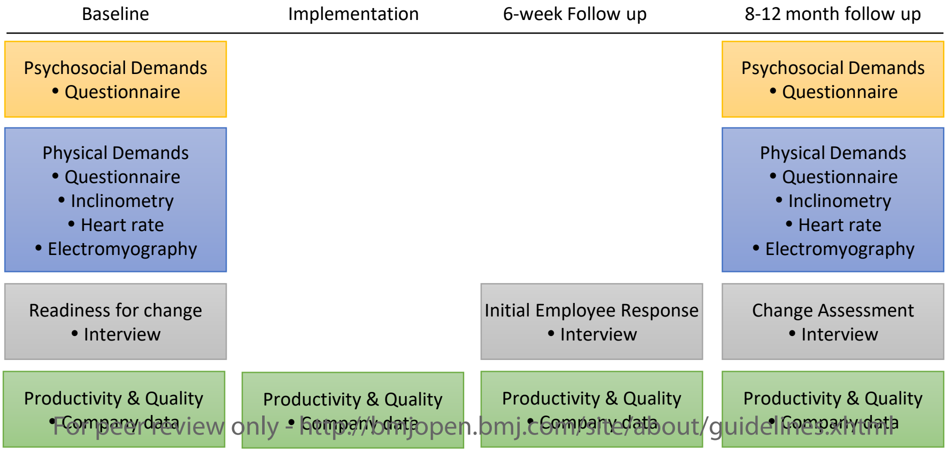

For peer review only - http://bmjopen.bmj.com/site/about/guidelines.xhtml

(a) Think about all parts of your job. Make a mark on each black line.

How physically demanding is your job?	Not at all 0	Maximum 10
How mentally demanding is your job?	Not at all 0	Maximum 10

(b)

	Always	Often	Some-times	Seldom	Never / Hardly ever
Do you have to work very fast?	○	○	○	○	○

For peer review only - http://bmjopen.bmj.com/site/about/guidelines.xhtml

BMJ Open

Assessing the efficacy of a job rotation for improving occupational physical and psychosocial work environment, musculoskeletal health, social equality, production quality, and resilience at a commercial laundromat: Protocol for a longitudinal case study.

Journal:	*BMJ Open*
Manuscript ID	bmjopen-2022-067633.R1
Article Type:	Protocol
Date Submitted by the Author:	24-Mar-2023

Complete List of Authors:	Jackson, Jennie; University of Gävle, Department of Occupational Health Sciences and Psychology<br>Sund, Marianne; Elis Textile Service AB<br>Barlari Lobos, Griztko ; Elis Textile Service AB<br>Melin, Lars; Elis Textile Service AB<br>Mathiassen, Svend ; University of Gavle, Occupational Health Sciences and Psychology

**Primary Subject Heading**:	Occupational and environmental medicine
Secondary Subject Heading:	Evidence based practice
Keywords:	OCCUPATIONAL & INDUSTRIAL MEDICINE, HEALTH ECONOMICS, Musculoskeletal disorders < ORTHOPAEDIC & TRAUMA SURGERY

SCHOLARONE™
Manuscripts

Assessing the efficacy of a job rotation for improving occupational physical and psychosocial work environment, musculoskeletal health, social equality, production quality, and resilience at a commercial laundromat: Protocol for a longitudinal case study.

Jennie A. Jackson[a*], Marianne Sund[b], Griztko Barlari Lobos[b], Lars Melin[b], Svend Erik Mathiassen[a]

a. Centre for Musculoskeletal Research, Department of Occupational Health Sciences and Psychology, University of Gävle, 801 76 Gävle, Sweden
b. Elis Textile Service AB, Hamrångevägen 21, 816 31 Ockelbo, Sweden

[*] corresponding author – jennie.jackson@hig.se.

For peer review only

ABSTRACT

Introduction: Job rotation is a work organization strategy used to reduce work-related exposures and musculoskeletal complaints, yet evidence for the efficacy of the approach is weak. Mismatch between job rotation and company needs, lack of full implementation, lack of exposure variation in included tasks, and failure to assess variation may underlie inconclusive research findings to date. The study aims to develop a job rotation with company stakeholders, perform a process evaluation of the implementation, and determine the extent to which the intervention improves the physical and psychosocial work environment, indicators of health, gender and social equality among workers, and production quality and resilience.

Methods and analysis: Approximately 60 production workers at a Swedish commercial laundromat will be recruited. Physical and psychosocial work environment conditions, health, productivity and gender and social equality will be assessed pre- and post- intervention using surveys, accelerometers, heart rate, electromyography and focus groups. A task-based exposure matrix will be constructed, and exposure variation estimated at the level of the individual worker pre- and post- intervention. An implementation process evaluation will be conducted. Job rotation efficacy will be assessed in terms of improvement in work environment conditions, health, gender and social inequality, and production quality and resilience. This study will provide novel information on the effects of the job rotation on physical and psychosocial work environment conditions, production quality and rate, health, and gender and social inequality among blue-collar workers in a highly multicultural workplace.

Ethics and dissemination: The study received approval from the Swedish Ethical Review Authority. Results of the project will be shared directly with the employees, managers and union representatives from the participating company, other relevant labour market stakeholders, and with researchers at national and international conferences and via scientific publication.

Trial registration: The study is pre-registered with the Open Science Framework (https://osf.io/zmdc8/).

Keywords: repetitive work, variation, work task organization, work rotation, job rotation, musculoskeletal health, intervention, implementation, co-creation, co-created program logic, process and outcome evaluation

For peer review only - http://bmjopen.bmj.com/site/about/guidelines.xhtml

STRENGTHS AND LIMITATIONS

- Co-creation participative intervention and study design
- Assessment of job rotation intervention effects on physical and psychosocial work environment conditions, health, production quality, and resilience with consideration to gender and social equality
- Process evaluation of the intervention implementation
- Consideration of inequality both from a male-female (gender) and a place of birth (ethnicity)
- Case study based on a limited number of workers at a single work site

For peer review only

For peer review only - http://bmjopen.bmj.com/site/about/guidelines.xhtml

INTRODUCTION:

Job rotation is an organization-level strategy that involves alternating workers between tasks that, ideally, differ in physical and psychosocial demands. In the field of ergonomics, job rotation has predominantly been used with the aim of reducing work-related exposures and musculoskeletal complaints; however, current evidence has been described as weak (1) or inconclusive (2) regarding the efficacy of job rotation to prevent musculoskeletal disorders (MSD). Recent work has even suggested that job rotation can increase the overall risk of workplace injury (3).

The efficacy of a job rotation is theoretically dependent on the extent to which the tasks included in the rotation differ and thus the extent to which more variation can be introduced in the jobs of individual workers rather than appearing between workers (4). Quantification of exposure *variation* is therefore required before and after job rotation to determine whether the intervention succeeded in altering exposure patterns. Mixed study results to date regarding the effects of job rotation on health outcomes may be the result of a lack of objective assessment of exposure variation both before and after intervention. In Leider's 2015 systematic review of evidence of the effects of job rotation on musculoskeletal complaints, only 4 of the 16 studies quantified aspects of both exposure and MSD health outcomes, and none of the studies assessed exposure variation (5–8).

Consideration of psychosocial factors is also paramount when considering the complex relationships between work organization, work environment and health (including MSDs) (9–13) – Figure 1. Similar to physical exposures, job rotation is generally undertaken to improve psychosocial conditions. While positive correlations have been shown for higher worker job satisfaction ratings (1), there is inconclusive evidence to date for the effects of job rotation on other psychosocial risk factors, for example, job control (14) or health outcomes, including work absence or sick leave (1). Again, mixed study results to date may be the result of low-quality study designs and the lack of assessment of exposure and outcome variables before and after intervention.

The conceptual model used in this study is given in Figure 1 and illustrates the interconnection of ergonomic and production factors and outcomes which warrant consideration when making changes in work organization. R*ationalization* is defined here as comprising changes made to *'maximize productivity, quality and efficiency under the prevailing conditions of, among other things legislation, education level of available work force, and culture'* (11). In addition to potential ergonomic gains, job rotation has been shown to improve employee skill/competence, productivity, quality (15,16), decrease worker turnover (15), and improve organizational flexibility (16). Thus, job rotation is *per se* a rationalization. At the time this paper was written, we were unable to find any studies that had simultaneously and quantitatively addressed the effects of a job rotation on the interrelated ergonomics and production factors and outcomes outlined in figure 1. The job rotation will be designed according to the Goldilocks Work approach (17,18) to distribute physical and mental loads during productive work to improve worker health and, to the extent possible, capacity. The job rotation will aim to distribute tasks with higher physical and/or mental demands across workers, and to schedule work to facilitate recovery by interspacing higher demand tasks with lower demand tasks.

insert Figure 1 around here

For peer review only - http://bmjopen.bmj.com/site/about/guidelines.xhtml

Inconclusive findings in previous job rotation studies may also be the result of a mismatch between specific company needs and the implemented job rotation (19,20). To improve intervention efficiency, a participatory approach has been called for in which interventions are co-created by researchers together with relevant company stakeholders, such as, management, health and safety, union, and production representatives (21,22). Co-creation can also increase knowledge within an organization regarding the problem(s) to be targeted by an intervention and how the intervention will address the problem(s). This can enhance readiness for change, which is a necessity for successful intervention implementation (23,24). Co-created program logic (COP) is a participatory approach that begins with a set of outcomes identified by stakeholders, around which a specific intervention is subsequently designed (25). COP is also a tool for defining, through negotiation between stakeholders and researchers, the outcomes to be measured and the optimal time points for the measurements. COP has proved successful in developing study aims and design and in improving readiness for change, intervention contextual fit, and implementation efficacy (23).

Insufficient process evaluation of the intervention implementation, including failure to fully implement the intervention (26), has also been identified as a common weakness in prior job rotation studies (27). Consideration of how job rotation has been implemented in sub-populations of workers may prove salient in fully understanding the implementation and in the effects of an intervention, but to date has been sparse. Findings from a job rotation intervention study at a grocery store found work tasks deemed worst by employees were excluded from the intervention and subsequently allotted to a group of female workers who were, thus, not included in the job rotation (28). This example is in line with the findings of a 2013 review report prepared for the Swedish Work Environment Authority, finding that task allocation within jobs was gendered, where female workers tended to be given more monotonous and repetitive tasks than male workers (29). Further, there is broad consensus in the literature that females receive less on-the-job training than men, including initial on-the-job training (30), which positions females to be assigned fewer tasks and possibly to be excluded for job rotation initiatives comprising tasks requiring more than basic training or competences. Foreign-born employees have also been shown to receive less on-the-job training than native born employees (31). Consideration of job rotation design and implementation within a social context may therefore be crucial. The many limitations of previous studies and the lack of research simultaneously considering aspects of social inequality in task assignment, job rotation, and health demonstrate the need for further research that considers the effects of job rotation from a wide range of perspectives.

The aim of this study is to develop a job rotation intervention at a commercial laundromat in concert with company stakeholders using co-created program logic, evaluate the process implementation of the job rotation intervention, and determine the extent to which the intervention can improve the physical and psychosocial work environment and indicators of health, advance gender and social equality among workers, and increase production quality and resilience without negatively affecting production rates.

The specific aims of the study are to:

1) Describe the co-creation program logic process used to develop the intervention and study design.
2) Provide a process evaluation for the implementation, including assessment of readiness for change and the extent to which the intervention was implemented.
3) Determine the extent to which the job rotation intervention increased exposure variation and lead to changes in physical and mental work environment factors and indicators of health.

For peer review only - http://bmjopen.bmj.com/site/about/guidelines.xhtml

4) Evaluate social and gender inequality in work organization, working conditions, and health before, during, and after the job rotation intervention
5) Assess the impact of the job rotation on production rate, quality and resilience.

METHODS AND ANALYSIS

The study will be performed at a commercial laundromat employing approximately 60 full time workers in laundry handling tasks. The study resulted from contact initiated by the study site: a commercial laundromat in Sweden. The laundromat management and union saw room for improvement in physical and psychosocial working conditions and issues of equality, and were interested in implementing a job rotation, but sought help to successfully make the change. We began by establishing a steering committee of eight people that comprised company management (LM, GBL), production workers, health and safety and union representatives (MS) and researchers (JJ, SEM) to jointly develop the study.

After extensive negotiations, the steering committee arrived at a co-created study design that included (i) development of an innovative job rotation intervention via co-creation with company stakeholders, (ii) evaluation of the implementation of the job rotation intervention, and (iii) assessment of the efficacy of the job rotation intervention using a pre-post intervention case study design. Methods for all three aspects of the study are presented below.

Development of the job rotation intervention using co-created program logic

The job rotation intervention was co-created in a collaboration between management (GBL), union representatives (MS) and researchers (JJ), and was guided by the Goldilocks Work approach principals (17,18) to find a health-promoting balance in both physical and mental work exposures. To better understand laundry processing tasks, one researcher (JJ) shadowed workers performing each task and, to the extent possible, spent 15-45 minutes performing each task. Company representatives and the researcher worked together to rate the physical and mental load (heavy/light) and the degree of repetitiveness (monotonous/variable) of each task. Led by the research team, company representatives grouped tasks into different proficiency levels, starting with a base group of tasks that they believed all workers could and should learn, and then moving onto tasks that required increased knowledge of production flow and task-specific training. Five hierarchical levels of tasks were created, namely: (i) basic tasks (which all workers could perform), (ii) driers and small machines, (iii) order picking and packing for hotel and medical customers, (iv) order picking and packing of work clothes, and (v) steering production flow.

The laundromat is physically divided into two sides – the *sorting* side, where bags of dirty laundry are received, emptied onto conveyor belts, and goods are sorted by colour and item prior to washing, and the *processing* side of the laundromat, where clean goods are dried, pressed, folded and packed. In general, rotation from sorting to processing is not typically done within a day as a shower is required before moving from sorting to processing. Base tasks were identified on both the sorting and processing sides, and so full-day rotation plans were established for each side to accommodate the hygiene requirements.

To meet company goals for improved language proficiency, social and gender equality and integration, the job rotation was based on *teams* comprising 3-6 workers that rotated as a unit. Teams were created to include at least 1 male and 1 female, 1 native Swede, ideally not more than 1

For peer review only - http://bmjopen.bmj.com/site/about/guidelines.xhtml

person with the same mother-tongue, and a mix of seniority levels in the competencies required for the tasks assigned to the team. A total of ten job rotation teams were formed; five of which performed basic tasks. Of these, four teams were assigned both sorting work and processing work, while one base group was assigned only processing work. Job training was required for nearly all workers to be able to perform all tasks assigned under the job rotation intervention. The company aimed to, in parallel, develop a new salary structure that would match the new proficiency 'steps' and competencies with employee wages. The company expected that the new work and wage structure would make for a more transparent development and promotion path, including making it easier for employees to understand and express interest in career advancement plans.

Implementation process and outcome evaluation

To assess the implementation process, semi-structured focus group interviews with each job rotation team and individual interviews with management will be conducted at three time points: immediately prior to the start of the job rotation intervention and at 6 weeks and 12 months follow-up. Data collected immediately prior to the job rotation intervention will be used to assess worker readiness for change and worker impressions of their new working schedules. Individual interviews with managers from this time point will be used to assess readiness to lead change. At 6-weeks follow-up, group interviews will be used to document the extent to which the job rotation intervention has been implemented during the initial phase and self-reported ability of workers to complete all assigned tasks. At 12 months follow-up, group interviews with each team will be used to document the progress and extent of implementation achieved. Individual interviews with management will be used to assess the company's perspective and experience of the implementation process and the extent of success. Together, the interviews will capture facilitators and barriers to the implementation process and will document additional changes occurring during the follow-up period that were un-related to the intervention, but which may have affected the way in which work was performed.

Interview data will be transcribed and analysed thematically (32), guided by research aim 2 to assess readiness for change and the extent of intervention implementation.

Assessment of job rotation intervention efficacy via pre-post study design

Using co-created program logic (COP) (25) as earlier outlined, outcome metrics were established by the researchers in concert with the company steering committee using an iterative procedure based on identifying desired changes that could directly be tied to the job rotation intervention. The study scientific testing protocol was then developed (JJ, SEM) and presented to the steering committee who completed a risk analysis prior to the protocol being approved.

Study design overview

The workers will be followed for approximately 12 months, with measurements occurring prior to, during, and 8-12 months following the job rotation intervention implementation (Figure 2). Baseline physical and psychosocial working conditions will be documented using (i) a questionnaire to assess the physical and psychosocial environments, (ii) technical measurements to assess posture, heart rate, and muscle activity levels during work, and (iii) semi-structured interviews to assess readiness for change. Initial response to the intervention will be assessed six weeks post implementation via semi-structured focus group interviews. At follow up, physical and psychosocial working conditions will be re-assessed using the baseline measurement protocol, and a final set of semi-structured focus

For peer review only - http://bmjopen.bmj.com/site/about/guidelines.xhtml

group interviews will be conducted to document production and working environment changes that occurred during the follow-up period but were unrelated to the job rotation intervention (33). Production rate and quality will be assessed weekly throughout the study period, using data collected by the company.

insert figure 2 around here

Study population

Permanent and contract workers currently employed in laundry handling tasks at the laundromat will be invited to participate in the study; the exact number of participants and inclusion criteria will vary by measurement type and research question, as outlined below. For the questionnaire, we will aim for a response rate of 70% (n = 35). For both technical measurement protocols we will collect data from all interested participants, and will aim for a minimum of 20 participants, which corresponds to approximately 30% of the total worker group. Focus groups interviews will be conducted with each team and will include all team members who elect to participate.

Approximately half of the employees are foreign born with varying levels of Swedish language proficiency, and there is a wide range in education levels across all employees. To ensure workers are informed, understand participation is voluntary and understand what participation entails, we will present all aspects of the research project in person to all employees using slides relying primarily on graphical displays of information, and we will provide live demonstration of technical measurement methods.

All production employees will be invited to participate in the study. Baseline survey response rates were 97% (n = 58), which suggests a strong likelihood for obtaining ample data for this case study throughout the follow-up period. Focus on developing and maintaining a strong relationship with the company has been present since study inception.

Pre- and post- job rotation measurements

Questionnaire

A custom questionnaire comprised of modules from well-established, validated and documented questionnaires and modules adapted from questionnaires previously used in our research group to assess other occupational groups (ex. (34,35) but adjusted to specifically capture information about the commercial laundromat environment. The modules in our questionnaire consider: personal and demographic parameters (9 questions), employment status and working hours (7 questions), specific work tasks currently performed and time spent per task over a week (6 questions), physical and mental demands of each work task and the overall job (4 questions with Borg CR-10 response scale (36)), self-rated work ability (4 questions), workload, fatigue and recovery (short SOFI questionnaire (37,38), 14 questions); musculoskeletal pain (Standardised Nordic questionnaire (39), 4 questions), job satisfaction (2 questions), and the psychosocial work environment (selected Copenhagen Psychosocial Questionnaire (COPSOQ) modules (40), 79 questions).

For peer review only - http://bmjopen.bmj.com/site/about/guidelines.xhtml

All employees will be invited to complete the questionnaire. The language and education level of the respondents was a focus during the development of all questionnaire modules, and substantial efforts were made to simplify language and response options in all custom modules. Where appropriate, a colour bar, ranging from green to red, was added to help participants understand the value (positive/neutral/negative) of the response options (figure 3a). We also added a colour bar to each COPSOQ question which helped to highlight that the response scales vary (positive to negative or negative to positive) by question (figure 3b).

insert figure 3 around here

To further assist employees, a workplace language course will be given one month prior to introducing the questionnaire for the 24 employees deemed to have the lowest language proficiency. As part of the course, a Swedish language teacher will review the questionnaire and teach employees how to respond to the different types of questions (short answer, multiple choice and CR-10 response scale). Lessons will be provided during working hours for groups of 8 employees at a time, be 1 hour in duration, and occur twice a week. In addition, all employees will be provided with a copy of the questionnaire one week in advance of the data collection.

Questionnaires will be completed during paid working hours in small groups (6-8 employees) sitting in the same room as a researcher. We will provide help as needed and encourage workers to join a group with other workers sharing the same mother tongue. Mobile phones and translation/dictionary apps will be permitted and encouraged. Workers will also be permitted to answer the survey at home if desired. The on-site researcher will have trained in giving consistent explanations. The same researcher will be present on all occasions.

Technical Measurements

Pre-implementation, technical data collection will be conducted to assess postures and heart rate over five consecutive days, and then to assess muscle activity levels during individual work tasks. All full-time permanent and contract employees working day shifts will be invited to participate in one or both technical data collections.

Post-implementation, we will evaluate any work tasks that have changed and any new work tasks that have been introduced.

Posture and heart rate assessment

Working postures, heart rate and heart rate variability will be assessed continuously over five consecutive working days. Postures will be assessed using wireless tri-axial accelerometers (Axivity, Newcastle upon Tyne, UK) affixed to the skin using double sided toupee tape (between the device and the skin) and breathable medical tape (over top of the device). Upper arm elevation will be recorded using units mounted according to 'LT INC' location (41): upper edge of the device aligned with the insertion of the deltoid muscle into the humerus, long axis aligned with the humerus. Trunk elevation angle will be recorded using a unit placed on the sternum with the top edge of the unit at the level of the sternoclavicular notch, and the long axis of the unit aligned with the line of gravity. A final accelerometer unit will be placed on the right thigh (long axis aligned with the femur) and will be used to determine activities (sit/stand/walk/run) during the five working days.

For peer review only - http://bmjopen.bmj.com/site/about/guidelines.xhtml

During the same 5-day period, participants will also wear a BodyGuard heart rate (HR) monitor (Firstbeat Technologies Oy, Finland) comprising two electrodes (Ambu, Denmark) positioned according to Firstbeat recommendations, a wireless logger (attached to the electrode positioned directly below the right clavicle), and a wire joining the two electrodes.

To permit task-level analysis of posture and heart rate, workers will record the specific tasks and times at which each task was performed over the 5-day period in a daily logbook. A researcher will briefly meet each worker individually once a day during the 5-day period. Placement and alignment of all accelerometer units will be verified and tape changed, as necessary, depending on worker discomfort and tape adhesion, and calibration exercises will be performed according to (41). The researcher will also confirm that the participant has been completing the work task log and responding to the fatigue assessment questions.

Muscle activity assessment

Muscle activity will be assessed using electromyography (EMG) recorded continuously during one working bout of approximately 2 hours, and during the adjacent coffee break (15 minutes) or lunch break (30 minutes). During a work bout, workers typically perform one task, but may perform two or more tasks. EMG will be recorded bilaterally from the trapezius (Tr EMG) and forearm (FA EMG) muscles using a portable logger and EMG sensors from Biometrics Ltd. (Newport, UK). At each recording site, the skin will be shaved and cleaned with alcohol prior to applying a disposable electrode (Ambu, Denmark). A single ground electrode (Ambu, Denmark) will be positioned atop a prominent thoracic vertebra (usually C7). Tr EMG electrodes will be positioned 2 cm lateral to the midpoint between C7 and the acromion process and aligned along the direction of the muscle fibres (42). Bilaterally, a 'through the forearm' assessment of FA EMG will be made, as described by (43), using electrodes placed just lateral to the muscle bellies of the flexor digitorum superficialis and extensor digitorum communis muscles. Given the physical nature of the laundry handling work tasks, the tight spaces through which workers are often required to navigate, and the heavy machinery involved in the work, care will be made to ensure the EMG recording equipment is securely attached to the participant and that the cables will not interfere with their work.

A rest file and three repeats of muscle-specific reference contractions will be collected to permit normalisation. For the trapezius muscles, a sub-maximal reference voluntary exertion (RVE) normalisation will be employed according to (Mathiassen, et al., 1995). RVE trials will be 15 s in duration (Åkesson et al., 1997; Hansson et al., 2000) interspaced by 30 s of rest. For the forearm, a maximum voluntary exertion (MVE) pinch grip normalisation task will be employed. Subjects will gradually increase their pinch grip on a pinch dynamometer over 3 seconds, until reaching and holding their MVE for 3 seconds before gently ramping down their exertion. MVE trials will be approximately 7 s in duration interspaced by 60 s of rest

A researcher will follow workers during the EMG recording period to document the times and tasks performed. Workers will also be videotaped during the EMG recording period to provide additional assistance in dividing the full EMG recording into component tasks.

Focus group interviews

Semi-structured focus group interviews will be conducted with each job rotation team and with individual managers at three time points and analysed as outlined in the 'Implementation process and outcome evaluation' section above. Information regarding facilitators and barriers to the

For peer review only - http://bmjopen.bmj.com/site/about/guidelines.xhtml

intervention and additional changes occurring during the follow-up period will be used to give additional context to the survey and technical measurement data.

Company data

The laundromat will track and provide weekly data on production rate (total tonnes laundered), quality, worker sick days, days off for workers to care for sick children, accidents, injuries and worker turnover from baseline until the end of the follow up period

Data processing and statistical analysis

Most previous studies documenting working conditions and occupational physical exposures via questionnaires and technical measurements have data analyses based on standard statistical methods, including a priori power calculations. Such methods assume that participants represent, in theory, the total population of all workers in similar settings. In our study, results consider a specific workplace and its 60 participant workers and not all workers employed in all commercial laundromats. We accept this notion in the research project and acknowledge it by adjusting basic descriptive statistics, such as SDs, by a finite population correction term (35). In terms of the finite population correction, the anticipated number of technical measurement participants corresponds to approximately 30% of the total worker population.

To estimate a study size necessary to obtain sufficient power for assessing significant differences requires information regarding the relevant and interesting effect sizes, however these data are not currently available for most of our quantitative variables. Further, information regarding the variance of the variables is also required but, again, these data are not currently available. Accordingly, in this case study of a single laundromat, we will aim to document changes according to our pre-post design.

Differences pre- to post- job rotation in work task allocation, psychosocial work environment conditions (including COPSOQ dimension) and self-reported health will be analysed, including consideration of confounders such as age, geographical location, marital status, family situation and other socioeconomic factors ultimately included in the questionnaires. Change scores will be calculated between baseline and follow-up for all COPSOQ dimensions.

The extent to which factors differed between males and females and between Swedish-born and foreign-born employees will, to the extent possible, be considered using a stratification approach.

The Firstbeat SPORTS uploader (V.1.0; Firstbeat Technologies) will be used for uploading and visually identified artefacts will be removed. Average heart rate (HR) during work, non-work and sleep will be calculated using a custom MATLAB script. Relative aerobic workload will be calculated in a previously developed custom MATLAB script and will be based on the heart rate reserve (HRR), which is a well-established estimate of the body’s workload and the body’s workload relative to individual fitness.

The accelerometry data will be processed using a custom-made MATLAB program to determine the time spent sitting, standing, walking, in light physical activity and in moderate to vigorous physical activity during both working- and non-working hours. Trunk and upper arm elevation data will be calibrated (41) to angles calculated using an in-house custom MATLAB program.

For peer review only - http://bmjopen.bmj.com/site/about/guidelines.xhtml

Accelerometry and HR data will be determined for each individual work task using the log book kept by workers. For each task, HR variables calculated will include HR intensity (relative aerobic workload, measured in terms of % HRR), time at different intensity levels, and HR variability, and postural variables, mean angle, time in neutral, and minute-to-minute variance of angle (44). Tasks where worker HR reaches or exceeds 60% HRR will be deemed sufficiently high-intensity for potentially generating increases in physical capacity (45).

Post collection, EMG signals will be Butterworth filtered (30 Hz high pass) for minimization of electrocardiogram (ECG) contamination (Drake and Callaghan, 2006), offset corrected, and root-mean-square (RMS) converted (100 ms moving window), then quadratically rest adjusted. All files will be visually inspected to ensure good data quality. The mean amplitude across a stable 10s period from each of the three RVE trials will be calculated and the mean of the three trials will be used to normalise the subject's Tr-EMG data. The largest 0.5 s mean EMG value will be determined across each MVE, and the largest single MVE will be used to normalise the subject's FA-EMG

EMG data from the approximately 2-hour work bout will be partitioned, if necessary, to individual work tasks using the time log kept by the researcher during data collection and, when necessary, assisted by the time stamp in the video data. For each task, the following muscle activity variables will be calculated using a custom-made MATLAB program: mean, peak, time at rest, minute-to-minute variance, and SULMA periods (46).

A task-based exposure matrix will be constructed comprising the posture and muscle activity variables, as outlined above. Using the log book data, individual exposure profiles will be constructed by estimating exposure variation for each day using an approach similar to the job variance ratios described by (44). Weekly variation will be assessed at the individual level by the mean variation across days and the between-days variance across the five days.

Pre-post changes in individual estimates of daily and weekly exposure variation will be assessed using repeated measures ANOVA analyses and, in cases where multiple variables reflect similar properties of exposure, using MANOVA analyses. ANOVA analyses will include a correction for the effects of multiple comparisons.

Interview data

To the extent possible, differences in experiences of the job rotation will be considered between male and female employees and Swedish- and foreign-born employees. Management interview data will be primarily used to assist in interpreting and understanding survey and technical data findings including changes occurring during the follow-up that could have influenced work organization or work environment unrelated to the intervention (33)(ref).

In interpreting focus group interviews Acker's theory of inequality regimes will be used as a framework for assessing how inequalities are produced and reproduced (47). Acker's theory comprises both organizational and individual levels and states that inequality regimes exist in all organizations, and can be defined as 'loosely interrelated practices, processes, actions and meanings' that create power orders from intersections of gender and ethnicity within the organization (47).

Patient and public involvement

For peer review only - http://bmjopen.bmj.com/site/about/guidelines.xhtml

No patient involved.

Registration

The study is pre-registered with the Open Science Framework (https://osf.io/zmdc8/).

Ethics and dissemination

The study received approval from the Swedish Ethical Review Authority (reference number 2019-00228). Written informed consent will be obtained for each data collection method, namely: accelerometry and heart rate monitoring, EMG, survey and focus group interview participation.

The results of the project will be shared directly with the Elis employees and managers at the participating laundromat, the worker's union (IF Metall) and the central Swedish and Global Elis-concerns via presentations and summary reports. Research results will also be disseminated via publication in international, peer-reviewed, open-access scientific journals and presentation at international scientific conferences.

For peer review only

Strengths and Limitations

The major novel contributions of this study to the literature are (i) the broad scope of the evaluation of the job rotation intervention, including consideration of the effects of the rationalization on work environment conditions, health, and production quality and resilience with consideration to gender and social equality and (ii) the inclusion of a process evaluation of the intervention implementation.

The consideration of inequality both from a male-female (gender) and a place of birth (ethnicity) perspective is in line with intersectionality theory (48) and further reflects the reality of modern immigration. It also reflects the Swedish government's latest Work Environment Strategy which prioritizes increased efforts towards sustainability with the goal of all employees (regardless of sex or place of birth) having a work environment that makes them able to, capable of, and willing to work until official retirement age (49).

The primary limitation of this longitudinal case study is the limited number of workers, and the inclusion of a single work site which will limit the generalizability of the results. Still, we anticipate that our findings on the impact of job rotation on physical and psychosocial work conditions, health and equality will be immediately useful for other laundromats in the global Elis concern. Further, we anticipate findings regarding social equality to be relevant for other commercial laundromats and small- and medium-sized production-based industries with high employee diversity. The study is also limited by the lack of information required to perform power calculations a priori (i.e. knowledge of the relevant/interesting effect sizes and size of variance for most of the quantitative variables). Upon completion of this study, these data full be available to guide future studies.

Funding

This work was supported by the Swedish Research Council for Health, Working Life and Welfare (Forte) grant number 2009-1761.

For peer review only - http://bmjopen.bmj.com/site/about/guidelines.xhtml

Authors' contributions

JJ and SEM conceptualized the project. All co-authors (JJ, MS. GBL, LM, SEM) participated in the co-creation process in which the study design and intervention were developed with JJ leading the process. All authors made substantial contributions to the development of the research study aims. JJ, MS and GBL developed the job rotation. JJ and SEM developed the research study design. JJ collected pilot data to assess task exposure variation.

All authors approve the submitted version and agree to be personally accountable for their contributions.

All authors read and approved the final manuscript

Acknowledgements

The authors wish to thank the production workers at the Elis laundromat in Ockelbo Sweden for generously welcoming the research team and embarking on this journey together with genuine enthusiasm.

Competing interests statement

The authors declare no competing interests.

For peer review only

Figure Titles

Figure 1: Conceptual model illustrating overlapping interests of production and ergonomics. *Proposed interactions are shown in arrows between rationalization (job rotation, in the current study) and work environment factors (shown in rectangles), and health and productivity outcomes (shown in ovals). Model adapted from* (11,12).

Figure 2: Study design

Figure 3: Examples of survey questions showing colour bar used to assist in understanding the value of response options. *(a) A custom module question and (b) A COPSOQ question.*

For peer review only

For peer review only - http://bmjopen.bmj.com/site/about/guidelines.xhtml

References

1. Padula RS, Comper MLC, Sparer EH, Dennerlein JT. Job rotation designed to prevent musculoskeletal disorders and control risk in manufacturing industries: A systematic review. Appl Ergon [Internet]. 2017;58:386–97. Available from: http://dx.doi.org/10.1016/j.apergo.2016.07.018

2. Leider PC, Boschman JS, Frings-Dresen MHW, van der Molen HF. Effects of job rotation on musculoskeletal complaints and related work exposures: a systematic literature review. Ergonomics. 2015;58(1):18–32.

3. Kuijer PPFM, Van Der Beek AJ, Van Dieën JH, Visser B, Frings-Dresen MHW. Effect of job rotation on need for recovery, musculoskeletal complaints, and sick leave due to musculoskeletal complaints: A prospective study among refuse collectors. Am J Ind Med. 2005;47(5):394–402.

4. Mathiassen SE. Diversity and variation in biomechanical exposure: What is it, and why would we like to know? Appl Ergon. 2006;37(4 SPEC. ISS.):419–27.

5. Fernström EAC, Åborg CM. Alterations in shoulder muscle activity due to changes in data entry organisation. Int J Ind Ergon. 1999;23(3):231–40.

6. Fredriksson K, Bildt C, Hägg G, Kilbom Å. The impact on musculoskeletal disorders of changing physical and psychosocial work environment conditions in the automobile industry. Int J Ind Ergon. 2001;28(1):31–45.

7. Rissén D, Melin B, Sandsjö L, Dohns I, Lundberg U. Psychophysiological stress reactions, trapezius muscle activity, and neck and shoulder pain among female cashiers before and after introduction of job rotation. Work Stress. 2002;16(2):127–37.

8. Arvidsson I, Balogh I, Hansson G åke, Ohlsson K, Åkesson I, Nordander C. Rationalization in meat cutting - Consequences on physical workload. Appl Ergon. 2012;

9. Bao SS, Kapellusch JM, Merryweather AS, Thiese MS, Garg A, Hegmann KT, et al. Relationships between job organisational factors, biomechanical and psychosocial exposures. Ergonomics. 2016;59(2):179–94.

10. Bongers PM, Kremer AM, Laak J Ter. Are psychosocial factors, risk factors for symptoms and signs of the shoulder, elbow, or hand/wrist?: A review of the epidemiological literature. Am J Ind Med. 2002;41(5):315–42.

11. Winkel J, Westgaard RH. Editorial: A model for solving work related musculoskeletal problems in a profitable way. Appl Ergon. 1996;27(2):71–7.

12. Rolander B, Jonker D, Winkel J, Sandsjö L, Balogh I, Svensson E, et al. Working conditions, health and productivity among dentists in Swedish public dental care - a prospective study during a 5-year period of rationalisation. Ergonomics. 2013;56(9):1376–86.

13. Sorensen G, McLellan DL, Sabbath EL, Dennerlein JT, Nagler EM, Hurtado DA, et al. Integrating worksite health protection and health promotion: A conceptual model for intervention and research. Prev Med (Baltim) [Internet]. 2016;91:188–96. Available from: http://dx.doi.org/10.1016/j.ypmed.2016.08.005

14. Balogh I, Ohlsson K, Hansson GÅ, Engström T, Skerfving S. Increasing the degree of automation in a production system: Consequences for the physical workload. Int J Ind Ergon. 2006;36(4):353–65.

15. Jorgensen M, Davis K, Kotowski S, Aedla P, Dunning K. Characteristics of job rotation in the

For peer review only - http://bmjopen.bmj.com/site/about/guidelines.xhtml

Midwest US manufacturing sector. Ergonomics. 2005;48(15):1721–33.

16. Cristini A, Pozzoli D. Workplace practices and firm performance in manufacturing: A comparative study of Italy and Britain. Int J Manpow. 2010;31(7):818–42.

17. Straker L, Mathiassen SE, Holtermann A. The “Goldilocks Principle”: designing physical activity at work to be “just right” for promoting health. Br J Sports Med. 2018;52(13):818–9.

18. Holtermann A, Mathiassen SE, Straker L. Promoting health and physical capacity during productive work: The goldilocks principle. Scand J Work Environ Heal. 2019;45(1):90–7.

19. Moore GF, Evans RE. What theory, for whom and in which context? Reflections on the application of theory in the development and evaluation of complex population health interventions. SSM - Popul Heal [Internet]. 2017;3(December 2016):132–5. Available from: https://doi.org/10.1016/j.ssmph.2016.12.005

20. Nielsen K, Randall R. Opening the black box: Presenting a model for evaluating organizational-level interventions. Eur J Work Organ Psychol. 2013;22(5):601–17.

21. Leask CF, Sandlund M, Skelton DA, Altenburg TM, Cardon G, Chinapaw MJM, et al. Framework, principles and recommendations for utilising participatory methodologies in the co-creation and evaluation of public health interventions. Res Involv Engagem [Internet]. 2019;5(1):2. Available from: https://doi.org/10.1186/s40900-018-0136-9

22. Von Thiele Schwarz U, Nielsen K, Edwards K, Hasson H, Ipsen C. How to design , implement and evaluate organizational interventions for maximum impact : the Sigtuna Principles. Eur J Work Organ Psychol [Internet]. 2021;30(3):415–27. Available from: https://doi.org/10.1080/1359432X.2020.1803960

23. Cedstrand E, Alvesson HM, Augustsson H, Bodin T, Bodin E, Nyberg A, et al. Co-creating an occupational health intervention within the construction industry in Sweden: Stakeholder perceptions of the process and output. Int J Environ Res Public Health. 2021;18(24).

24. Billsten J, Fridell M, Holmberg R, Ivarsson A. Journal of Substance Abuse Treatment Organizational Readiness for Change (ORC) test used in the implementation of assessment instruments and treatment methods in a Swedish National study. J Subst Abuse Treat [Internet]. 2018;84:9–16. Available from: https://doi.org/10.1016/j.jsat.2017.10.004

25. von Thiele Schwarz U, Richter A, Hasson H. Getting everyone on the same page: Co-created program logic (COP). In: Nielsen K, Noblet A, editors. Organizational Interventions for Health and Well-being A Handbook for Evidence-Based Practice. First. Exeter, Devon: Routledge; 2018. p. 42–67.

26. Murta SG, Sanderson K, Oldenburg B. Process evaluation in occupational stress management programs: A systematic review. Am J Heal Promot. 2007;21(4):248–54.

27. Rasmussen CDN. Is job rotation effective for prevention of work-related musculoskeletal disorders and sick leave? Occup Environ Med. 2017;74(8):543–4.

28. Johansson K, Fältholm Y, Abrahamsson L. Job Rotation Meets Gendered and Routine Work in Swedish Supermarkets. NORA - Nord J Fem Gend Res. 2015;23(2):109–24.

29. Lewis C, Mathiassen S. Belastning, genus och hälsa i arbetslivet. Rapport 2013:9. Stockholm; 2013.

30. Grönlund A. On-the-Job Training - A Mechanism for On-the-Job Training Investments Segregation ? Examining the Relationship between Gender , Occupation , and On-the-Job Training Investments. Eur Sociol Rev. 2012;28(3):408–20.

For peer review only - http://bmjopen.bmj.com/site/about/guidelines.xhtml

31. Barrett A, McGuinness S, O'Brien M, O'Connell P. Immigrants and Employer-provided Training. J Labor Res. 2013;34(1):52–78.

32. Braun V, Clarke V. Qualitative Research in Psychology Using thematic analysis in psychology Using thematic analysis in psychology. Qual Res Psychol [Internet]. 2006;3(2):77–101. Available from: http://www.tandfonline.com/action/journalInformation?journalCode=uqrp20%5Cnhttp://www.tandfonline.com/action/journalInformation?journalCode=uqrp20

33. Edwards K, Winkel J. A method for effect modifier assessment (EMA) in ergonomic intervention research. Appl Ergon [Internet]. 2018;72(May):113–20. Available from: https://doi.org/10.1016/j.apergo.2018.05.007

34. Bergsten EL, Mathiassen SE, Vingård E. Psychosocial work factors and musculoskeletal pain: A cross-sectional study among swedish flight baggage handlers. Biomed Res Int. 2015;2015.

35. Mathiassen SE, Bolin M, Olofsdotter G, Johansson E. Equal health at work? Protocol for an observational study of work organisation, workload and musculoskeletal complaints among women and men in grocery retail. BMJ Open. 2020;10(1):e032409.

36. Borg G. Psychophysical bases of perceived exertion. Med Sci Sport Exerc. 1982;14:377–81.

37. Åhsberg E. Dimensions of fatigue in different working populations. Scand J Psychol [Internet]. 2000 Sep 1;41(3):231–41. Available from: https://doi.org/10.1111/1467-9450.00192

38. Mathiassen SE, Hallman DM, Lyskov E, Hygge S. Can cognitive activities during breaks in repetitive manual work accelerate recovery from fatigue? A controlled experiment. PLoS One. 2014;9(11).

39. Kuorinka I, Jonsson B, Kilbom A, Vinterberg H, Biering-Sørensen F, Andersson G, et al. Standardised Nordic questionnaires for the analysis of musculoskeletal symptoms. Appl Ergon [Internet]. 1987;18(3):233–7. Available from: http://www.sciencedirect.com/science/article/pii/000368708790010X

40. Pejtersen JH, Kristensen TS, Borg V, Bjorner JB. The second version of the Copenhagen Psychosocial Questionnaire. Scand J Public Health. 2010;38(SUPPL. 3):8–24.

41. Jackson JA, Mathiassen SE, Wahlström J, Liv P, Forsman M. Is what you see what you get? Standard inclinometry of set upper arm elevation angles. Appl Ergon. 2015;47:242–52.

42. Mathiassen SE, Winkel J, Hägg GM. Normalization of surface EMG amplitude from the upper trapezius muscle in ergonomic studies—a review. J Electromyogr Kinesiol. 1995;5(4):197–226.

43. Takala EP, Toivonen R. Placement of forearm surface EMG electrodes in the assessment of hand loading in manual tasks. Ergonomics. 2013;56(7):1159–66.

44. Barbieri DF, Srinivasan D, Mathiassen SE, Nogueira HC, Oliveira AB. The ability of non-computer tasks to increase biomechanical exposure variability in computer-intensive office work. Ergonomics [Internet]. 2015;58(1):50–64. Available from: http://dx.doi.org/10.1080/00140139.2014.965753

45. Gormley SE, Swain DP, High R, Spina RJ, Dowling EA, Kotipalli US, et al. Effect of intensity of aerobic training on V̇O 2max. Med Sci Sports Exerc. 2008;40(7):1336–43.

46. Østensvik T, Veiersted KB, Nilsen P. A method to quantify frequency and duration of sustained low-level muscle activity as a risk factor for musculoskeletal discomfort. J Electromyogr Kinesiol [Internet]. 2009;19(2):283–94. Available from: http://dx.doi.org/10.1016/j.jelekin.2007.07.005

For peer review only - http://bmjopen.bmj.com/site/about/guidelines.xhtml

47. Acker J. Inequality regimes: Gender, class, and race in organizations. Gend Soc. 2006;20(4):441–64.

48. Mullings L, Schulz AJ. Gender, race, class, and health: Intersectional approaches. Jossey-Bass; 2006.

49. Regeringen. En god arbetsmiljö för framtiden –regeringens arbetsmiljöstrategi 2021–2025. Skr. 2020/21:92. Stockholm; 2021.

For peer review only

For peer review only - http://bmjopen.bmj.com/site/about/guidelines.xhtml

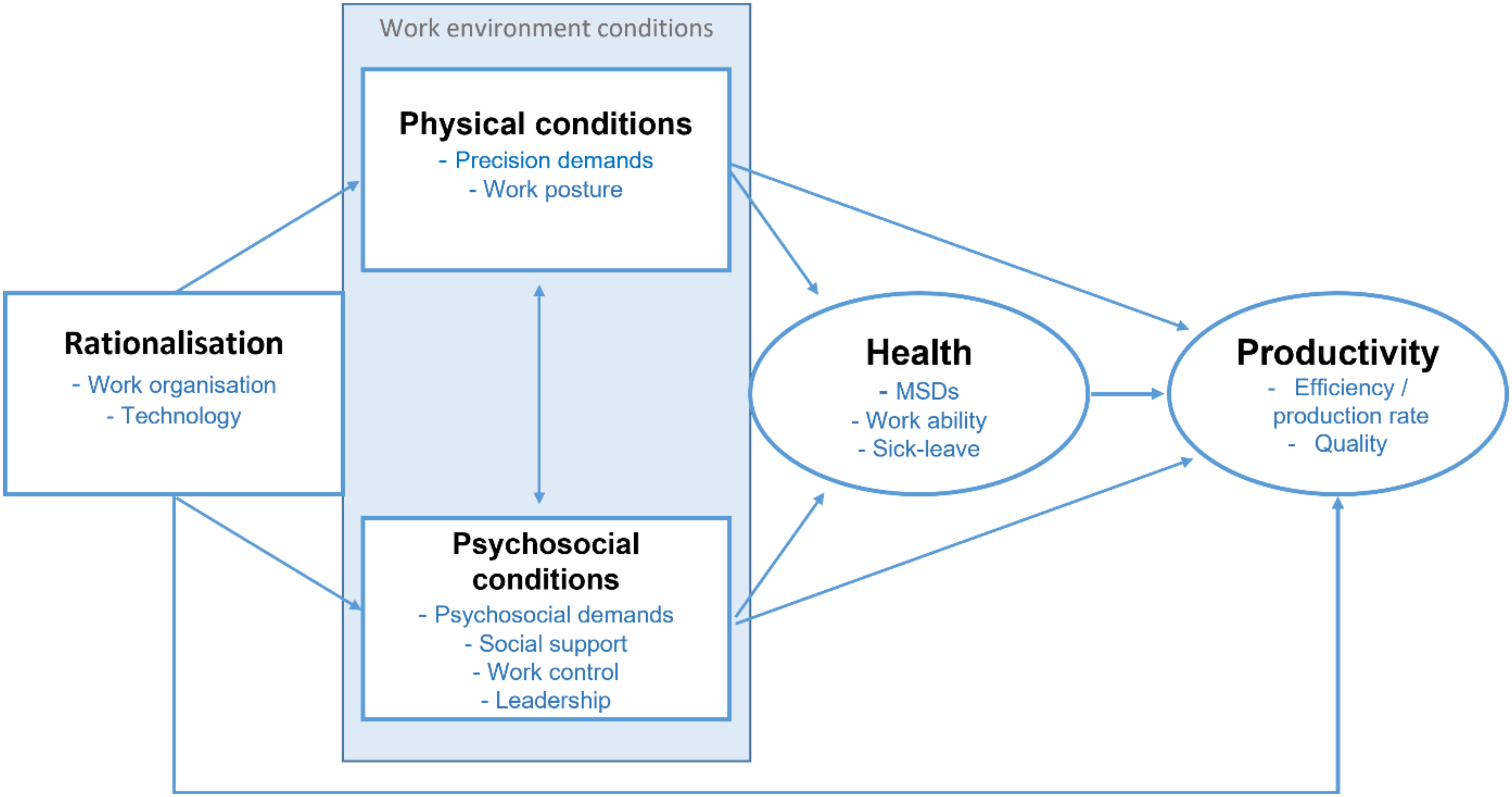
Work environment conditions
Physical conditions
- Precision demands
- Work posture
Rationalisation
- Work organisation
- Technology
Psychosocial conditions
- Psychosocial demands
- Social support
- Work control
- Leadership
Health
- MSDs
- Work ability
- Sick-leave
Productivity
- Efficiency / production rate
- Quality

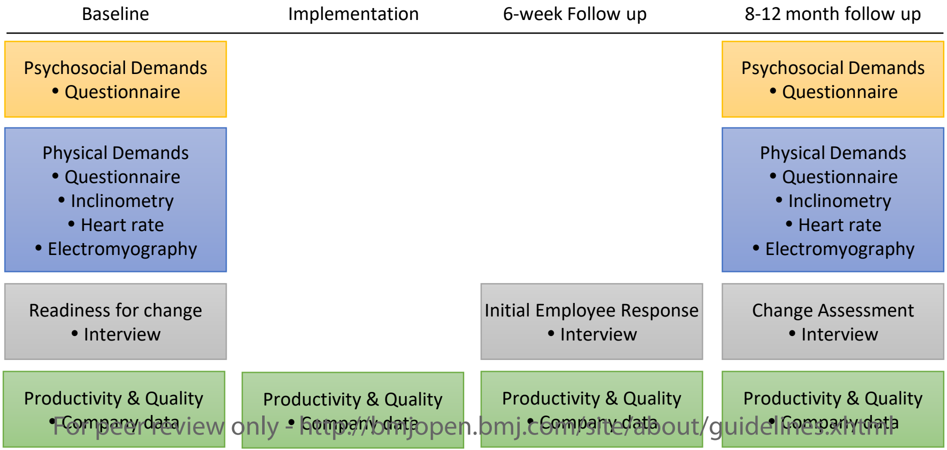

For peer review only - http://bmjopen.bmj.com/site/about/guidelines.xhtml

(a) Think about all parts of your job. Make a mark on each black line.

How physically demanding is your job?	Not at all 0	Maximum 10
How mentally demanding is your job?	Not at all 0	Maximum 10

(b)

	Always	Often	Some-times	Seldom	Never / Hardly ever
Do you have to work very fast?	○	○	○	○	○

For peer review only - http://bmjopen.bmj.com/site/about/guidelines.xhtml

STROBE Statement—checklist of items that should be included in reports of observational studies

	Item No.	Recommendation	Page No.	Relevant text from manuscript
**Title and abstract**	1	(*a*) Indicate the study's design with a commonly used term in the title or the abstract	1	Protocol for a longitudinal case study.
		(*b*) Provide in the abstract an informative and balanced summary of what was done and what was found	2	See abstract
**Introduction**				
Background/rationale	2	Explain the scientific background and rationale for the investigation being reported	4-5	
Objectives	3	State specific objectives, including any prespecified hypotheses	5-6	
**Methods**				
Study design	4	Present key elements of study design early in the paper	6	i) development of an innovative job rotation intervention via co-creation with company stakeholders, (ii) evaluation of the implementation of the job rotation intervention, and (iii) assessment of the efficacy of the job rotation intervention using a pre-post intervention case study design.
Setting	5	Describe the setting, locations, and relevant dates, including periods of recruitment, exposure, follow-up, and data collection	6-7	The study will be performed at a commercial laundromat employing approximately 50 full time workers in laundry handling tasks.

For peer review only - http://bmjopen.bmj.com/site/about/guidelines.xhtml
For peer review only

				The workers will be followed for approximately 12 months, with measurements occurring prior to, during, and 8-12 months following the job rotation intervention implementation (Figure 2).
Participants	6	(*a*) *Cohort study*—Give the eligibility criteria, and the sources and methods of selection of participants. Describe methods of follow-up<br>*Case-control study*—Give the eligibility criteria, and the sources and methods of case ascertainment and control selection. Give the rationale for the choice of cases and controls<br>*Cross-sectional study*—Give the eligibility criteria, and the sources and methods of selection of participants		
		(*b*) *Cohort study*—For matched studies, give matching criteria and number of exposed and unexposed<br>*Case-control study*—For matched studies, give matching criteria and the number of controls per case	7	Pre-post case study design – workers are own controls
Variables	7	Clearly define all outcomes, exposures, predictors, potential confounders, and effect modifiers. Give diagnostic criteria, if applicable	7-10	
Data sources/ measurement	8*	For each variable of interest, give sources of data and details of methods of assessment (measurement). Describe comparability of assessment methods if there is more than one group	*7-10*	*Questionnaire*<br>*Technical Measurements*<br>*Focus group interviews*
Bias	9	Describe any efforts to address potential sources of bias	7,9	All employees will be invited to participate<br>The language and education level of the respondents was a focus during the development of all questionnaire modules, and substantial efforts

For peer review only

				were made to simplify language and response options in all custom modules
Study size	10	Explain how the study size was arrived at	11	In our study, results consider a specific workplace and its 60 participant workers and not all workers employed in all commercial laundromats. We accept this notion in the research project and acknowledge it by adjusting basic descriptive statistics, such as SDs, by a finite population correction term (35). In terms of the finite population correction, the anticipated number of technical measurement participants corresponds to approximately 30% of the total worker population.

Continued on next page

For peer review only

Quantitative variables	11	Explain how quantitative variables were handled in the analyses. If applicable, describe which groupings were chosen and why	11
Statistical methods	12	(*a*) Describe all statistical methods, including those used to control for confounding	11
		(*b*) Describe any methods used to examine subgroups and interactions	11
		(*c*) Explain how missing data were addressed	Protocol study – N/A
		(*d*) *Cohort study*—If applicable, explain how loss to follow-up was addressed<br>*Case-control study*—If applicable, explain how matching of cases and controls was addressed<br>*Cross-sectional study*—If applicable, describe analytical methods taking account of sampling strategy	
		(*e*) Describe any sensitivity analyses	
**Results -**			**Protocol study – N/A**
Participants	13*	(a) Report numbers of individuals at each stage of study—eg numbers potentially eligible, examined for eligibility, confirmed eligible, included in the study, completing follow-up, and analysed	
		(b) Give reasons for non-participation at each stage	
		(c) Consider use of a flow diagram	
Descriptive data	14*	(a) Give characteristics of study participants (eg demographic, clinical, social) and information on exposures and potential confounders	
		(b) Indicate number of participants with missing data for each variable of interest	
		(c) *Cohort study*—Summarise follow-up time (eg, average and total amount)	
Outcome data	15*	*Cohort study*—Report numbers of outcome events or summary measures over time	
		Case-control study—Report numbers in each exposure category, or summary measures of exposure	
		Cross-sectional study—Report numbers of outcome events or summary measures	
Main results	16	(*a*) Give unadjusted estimates and, if applicable, confounder-adjusted estimates and their precision (eg, 95% confidence interval). Make clear which confounders were adjusted for and why they were included	
		(*b*) Report category boundaries when continuous variables were categorized	
		(*c*) If relevant, consider translating estimates of relative risk into absolute risk for a meaningful time period	

Continued on next page

For peer review only

Other analyses	17	Report other analyses done—eg analyses of subgroups and interactions, and sensitivity analyses		
**Discussion**				**Protocol study – N/A**
Key results	18	Summarise key results with reference to study objectives		
Limitations	19	Discuss limitations of the study, taking into account sources of potential bias or imprecision. Discuss both direction and magnitude of any potential bias		
Interpretation	20	Give a cautious overall interpretation of results considering objectives, limitations, multiplicity of analyses, results from similar studies, and other relevant evidence		
Generalisability	21	Discuss the generalisability (external validity) of the study results		
**Other information**				
Funding	22	Give the source of funding and the role of the funders for the present study and, if applicable, for the original study on which the present article is based	13	Forte grant number 2009-1761

*Give information separately for cases and controls in case-control studies and, if applicable, for exposed and unexposed groups in cohort and cross-sectional studies.

Note: An Explanation and Elaboration article discusses each checklist item and gives methodological background and published examples of transparent reporting. The STROBE checklist is best used in conjunction with this article (freely available on the Web sites of PLoS Medicine at http://www.plosmedicine.org/, Annals of Internal Medicine at http://www.annals.org/, and Epidemiology at http://www.epidem.com/). Information on the STROBE Initiative is available at www.strobe-statement.org.

For peer review only
